# Acute effects of outdoor and indoor walking on cigarette cravings, withdrawal symptoms and affective response during temporary smoking abstinence

**DOI:** 10.1007/s00213-023-06506-4

**Published:** 2023-11-25

**Authors:** Stefanie E. Schöttl, Kathrin Insam, Anika Frühauf, Prisca Kopp-Wilfling, Bernhard Holzner, Martin Kopp

**Affiliations:** 1 Department of Sport Science, University of Innsbruck, Fürstenweg 185, 6020 Innsbruck, Austria; 2 Department of Psychiatry, Psychotherapy and Psychosomatics, Psychiatry I, Medical University of Innsbruck, Anichstraße 35, 6020 Innsbruck, Austria

**Keywords:** Smoking, Craving, Withdrawal symptoms, Affective response, Well-being, Exercise, Physical activity, Sport

## Abstract

**Rationale:**

Cigarette smoking is one of the leading preventable causes of premature death worldwide. There is evidence in the literature that brief exercise units indoors can improve well-being in temporarily abstinent smokers and reduce cigarette cravings and withdrawal symptoms.

**Objective:**

Because exercise in natural environments showed enhanced psychological effects, the aim of our study was to examine the acute effects of outdoor exercise compared with indoor exercise on craving, withdrawal symptoms and affective response in temporarily abstinent smokers.

**Methods:**

In a randomized controlled within-subject-design, temporarily abstinent smokers (*N* = 16) participated in three interventions lasting 10 min: outdoor walking (OUT-EX), indoor walking (IN-EX) and a sedentary control condition (CC). Self-reported cigarette craving, withdrawal symptoms and affective response were assessed pre-, mid-, post-interventions and at follow-up.

**Results:**

In contrast to CC, OUT-EX and IN-EX significantly reduced cigarette cravings during and at the end of the intervention compared to pre-intervention, but not at 20 min follow-up. Cigarette withdrawal symptoms decreased significantly over time in all three groups, but no significant group differences were found. OUT-EX and IN-EX, but not CC, showed significantly improved affective valence at the end of the intervention and at follow-ups. Outdoor walking resulted in significantly lower cigarette cravings than indoor walking at the end of the intervention.

**Conclusion:**

The study adds to existing evidence that short bouts of indoor or outdoor exercise can help reduce cigarette cravings and increase well-being in abstinent smokers. Further studies are needed to address the potential additional effect of outdoor exercise on craving, affective states and smoking cessation.

## Introduction

Despite a decline in cigarette smoking rates over the last decades, tobacco use, especially cigarette smoking, remains one of the largest preventable causes of premature deaths worldwide (West 2017). More than 8 million people worldwide and about 780 000 people in the European Union (EU) died in 2019 as a result of tobacco consumption (World Health Organization 2021, OECD and European Union 2022). Cigarette smoking is associated with an increased risk of cardiovascular diseases, respiratory diseases and different types of cancer (Taghizadeh et al. 2016; Lariscy et al. 2018). However, quitting smoking can significantly reduce the risk of serious diseases and can increase life expectancy (Daniel et al. 2004; Jha 2020; Benowitz et al. 2023). A prospective study with more than 1 million women in the UK showed that smoking cessation before age 40 can avoid more than 90% of excess mortality caused by continuing smoking (Pirie et al. 2013). In addition, Crispo et al. (2004) found a more than 90% reduction in lung cancer risk among men in EU countries such as the UK, Germany and Sweden if they quit smoking before age 40.

However, the highly addictive nature of cigarettes makes it difficult to quit smoking (Jha 2020; Cheung et al. 2020). Nicotine is one of the most addictive ingredients in cigarettes that can cause dependence (Jha 2020). While smoking a cigarette, nicotine reaches the brain within 10 to 20 s and binds to acetylcholine receptors (nACHRs), which subsequently leads to the release of various neurotransmitters such as dopamine, noradrenaline, serotonin and ß-endorphin (Yildiz 2004; Yingst et al. 2022; West 2017). Regular cigarette consumption causes the development and reinforcement of tolerance to the effects of nicotine (Jha 2020). If the constant stimulation of the reward system by nicotine is absent, a strong craving to smoke and withdrawal symptoms such as sleep disturbances, difficulty concentrating, increased appetite, weight gain, restlessness or negative affect (e.g. depression, irritability) may occur (Yingst et al. 2022; Cheung et al. 2020; Weinberger et al. 2016; Robinson et al. 2019). Withdrawal symptoms after smoking cessation are therefore often the reason why smoking cessation quit attempts end in relapse within the first weeks (Weinberger et al. 2016; Yingst et al. 2022; Schnoll et al. 2011). Koçak et al. (2015) reported a relapse rate of over 50% one year after participating in a smoking cessation program. Moreover, unaided quit attempts are more likely to relapse than aided attempts involving, e.g. behavioural support (Perski et al. 2022).

A wide range of efficacious smoking cessation interventions such as pharmacological treatments including bupropion, varenicline and nicotine replacement therapy (transdermal patch, chewing gum, nasal or mouth spray, inhalator etc.), behavioural counseling and psychosocial support as well as newer approaches and strategies such as electronic cigarettes and smoking cessation apps can help to quit smoking and increase smoking cessation success (Aubin et al. 2014; West 2017; Abrantes et al. 2018; Xu et al. 2022; Abroms et al. 2013; Conklin et al. 2017; Hartmann-Boyce et al. 2022).

There is also some evidence that exercise as an adjunctive treatment for smoking cessation has many benefits (Daniel et al. 2004; Ussher et al. 2019). Exercise is a cost-effective treatment that is easy to access (Klinsophon et al. 2017). While exercising, attention is mainly focused on doing sports and smokers can be distracted from cravings for a cigarette (Ussher et al. 2019; Klinsophon et al. 2022). Regular exercise has not only a positive impact on the cardiovascular and respiratory system, but can also improve mood, well-being and can reduce symptoms for depression and anxiety (Darabseh et al. 2022; Huang et al. 2023). Furthermore, exercise in general can increase self-esteem and self-confidence, two positive attributes that can facilitate smoking cessation (Loprinzi et al. 2015). Weight gain following smoking cessation can also be reduced by exercising (Farley et al. 2012; Haasova et al. 2013). In addition, exercise, including short bouts of exercise, has positive effects on the reduction of cravings and withdrawal symptoms (Haasova et al. 2013; Roberts et al. 2012; Taylor et al. 2007; Ledochowski et al. 2013).

Several studies have shown that cigarette cravings in temporarily abstinent smokers were reduced during and/or after short bouts of exercise (Allen et al. 2018; Conklin et al. 2017; Abrantes et al. 2018; Daniel et al. 2004, 2007; Elibero et al. 2011; Everson et al. 2008; Hatzigeorgiadis et al. 2016; Jeffries et al. 2020; Roberts et al. 2015; Scerbo et al. 2010). A decline in withdrawal symptoms after brief exercise sessions compared to passive control conditions could also be observed in several studies (Allen et al. 2018; Conklin et al. 2017; Daniel et al. 2004, 2007; Everson et al. 2008). Moreover, Faulkner et al. (2010), Hatzigeorgiadis et al. (2016), Kurti and Dallery (2014) and Taylor and Katomeri (2007) reported increased delays in smoking the first cigarette after a short bout of exercise compared to a passive control condition in temporarily abstinent smokers. In the studies of Abrantes et al. (2018), Allen et al. (2018), Conklin et al. (2017), Elibero et al. (2011) and Everson et al. (2008), enhancement of mood and positive affect and reduction of negative affect during or after short exercise sessions were also discovered.

However, it is important to consider that the type, duration and intensity of exercise sessions differ across the studies in the literature and that different exercise characteristics may have a different impact on cigarette craving and withdrawal symptoms (Haasova et al. 2016; Darabseh et al. 2022). Besides aerobic exercise such as walking and running on a treadmill (Scerbo et al. 2010; Conklin et al. 2017) and cycling on an ergometer (Allen et al. 2018; Everson et al. 2008), strengthening exercise (resistance exercise) (Ho et al. 2014) as well as relaxation exercise (yoga) (Jeffries et al. 2020; Elibero et al. 2011) was used for a brief exercise unit. The short exercise sessions lasted from 5 min (Daniel et al. 2004) to 10–15 min (Faulkner et al. 2010; Scerbo et al. 2010) to over 30 min (Jeffries et al. 2020; Hatzigeorgiadis et al. 2016). In several studies, the influence of different exercise intensities on craving, withdrawal symptoms and affect was investigated (Haasova et al. 2016; Roberts et al. 2015; Scerbo et al. 2010; Janse van Rensburg et al. 2013; Daniel et al. 2004; Everson et al. 2008; Kurti and Dallery 2014). Daniel et al. (2004) reported a significant reduction in craving during and 5 min after moderate intensity exercise (stationary cycling) and a reduction in withdrawal symptoms 5 and 10 min post-exercise compared to light intensity exercise and a passive control condition. Kurti and Dallery (2014) also mentioned a greater reduction in cigarette craving after a moderate intensity exercise session compared to low intensity exercise and passive control condition. In the study of Janse van Rensburg et al. (2013), a significant increase in positive affect after vigorous exercise on a treadmill compared to passive control condition was observed but not after light intensity exercise session.

It should be noted, that in the previously mentioned studies, the short bouts of exercise took place in indoor settings. However, additional health benefits of exercise in natural environments (green exercise) were mentioned in the literature (Klaperski et al. 2019). There is growing evidence that exercise in natural environments may show enhanced effects on improved well-being, mood, self-esteem and reduced anxiety and stress compared to indoor exercise (Frühauf et al. 2016; Niedermeier et al. 2017; Klaperski et al. 2019; Pasanen et al. 2018; Barton and Pretty 2010). This was mostly evaluated in psychiatric (e.g. depression) and healthy populations (Frühauf et al. 2016; Niedermeier et al. 2017; Thompson Coon et al. 2011; Wicks et al. 2022), however has never been investigated in smokers regarding potential beneficial effects of outdoor exercise on craving symptoms. Therefore, the aim of our study was to investigate the acute effects of a short bout of outdoor and indoor walking compared to a sitting control condition on cigarette cravings, withdrawal symptoms, affective response and time to first cigarette in temporarily abstinent smokers.

## Method

### Design

The current study used a randomized control within-subject-design. All participants took part in three interventions: outdoor exercise (OUT-EX), indoor exercise (IN-EX), and a control condition (CC). The order of sessions was randomized across the test persons.

### Participants

Participants were recruited via flyers, social media and the University mail service. To be included in the study, male and female smokers had to be at least 18 years old and smoked ten cigarettes or more per day for at least the last two years. The eligibility for moderate physical activity determined by the Physical Activity Readiness Questionnaire (Thomas et al. 1992) was a further inclusion criteria. Exclusion criteria were persons under psychiatric or medical treatment and pregnant women.

### Sample size calculation

To determine the minimum number of participants to be included in the study, sample size was calculated (G*Power 3.1.9.7 (Faul et al. 2007)). The means and standard deviations between pre-test and post-intervention of an exercise group in the study of Taylor et al. (2005) were used to calculate the effect size. For a within-subject design with a power of 0.98 and alpha of 0.05, it was estimated that a sample of 15 participants are sufficient to detect differences. Sixteen of 18 recruited participants completed all measurements, as two test persons were excluded from the study due to illness and non-compliance with the nicotine abstinence before the measurements.

### Procedure

Approval by the local board for ethical questions in science of the University of Innsbruck was obtained and all participants signed an informed consent prior to the study. Participants were asked to refrain from smoking at least 10 h before the survey. The nicotine abstinence was controlled and noted by questioning the attendees. Following brief information, instructions and baseline measures, participants were randomly assigned to one of the three interventions (OUT-EX, IN-EX, CC). For randomization, the computer software Research Randomizer (Urbaniak and Plous 2013) was used. The interventions were carried out from mid-January to mid-February. Each participant was tested on three different days at the same time in the morning (early-late), with one week between each measurement.

### Interventions

During the indoor exercise sessions (IN-EX), the test persons walked on a treadmill without elevation. They were allowed to reduce or increase the speed on the treadmill. The IN-EX took place in Innsbruck (Tyrol) in a laboratory without a view of the outside and less natural light. During the outdoor exercise (OUT-EX) unit, participants walked on flat gravel paths in urban green space. To not influence the freely chosen walking speed of the test persons, the study leader walked behind the participants. In both exercise interventions, subjects were instructed to briskly walk as if they are too late for an appointment, but not to the point of breathlessness. In the control condition (CC), participants had to sit quietly without reading or using the smartphone. The interactions between the study leader and the participants were reduced to a minimum. To check that the intensity of the indoor and outdoor exercises is similar, heart rate was measured by Polar watches and perceived exertion was rated by participants using the Borg Scale (from 6 = extremely light to 20 = extremely hard) (Borg 1998).

### Assessment instruments

Baseline data were collected for sex, age, height, weight, nicotine dependence, readiness for physical activity and levels of physical activity. The participants also indicated the average number of cigarettes per day and how many years they have been smoking. For all interventions, the same questionnaires and measurements had to be completed at eight different assessment points: immediately prior to the interventions, during the interventions after 2, 4, 6 and 8 min, end of the interventions (after 10 min), and 10- and 20-min post-treatment. Table 1 shows the assessment instruments used at different time points. After each session, test persons had to inform the study leader via SMS or Email when they smoked the first cigarette.

**Table 1:** Assessment instruments used at the different time points

Prior to interventions	During interventions (after 2, 4, 6, 8 min.)	End of interventions (after 10 min.)	1. Follow-up (after 20 min.)	2. Follow-up (after 30 min.)
SoD	SoD	SoD	−	−
QSU	−	QSU	−	QSU
MPSS	−	MPSS	MPSS	MPSS
FS	FS	FS	FS	FS
FAS	FAS	FAS	FAS	FAS

*SoD*, strength of desire to smoke; *QSU*, Questionnaire of Smoking Urges; *MPSS*, Mood and Physical Symptoms Scale; *FS*, Feeling Scale; *FAS*, Felt Arousal Scale

#### Physical activity readiness questionnaire (PAR-Q)

To ensure that the smokers have the necessary physical condition to participate in the study, the physical activity readiness questionnaire (PAR-Q) had to be completed in advance (Thomas et al. 1992). The PAR-Q is a self-screening tool with seven questions about health designed to determine whether individuals are able to engage in physical activity (Bredin et al. 2013).

#### Subjective physical activity

To assess physical activity, the Godin-Shephard Leisure-Time Physical Activity Questionnaire (GSLTPAQ) was used (Godin 2011). Average weekly frequencies (minutes and amount) of strenuous, moderate and mild activities were self-reported by the participants. To obtain a leisure score index, each frequency score has to be multiplied by a corresponding Metabolic Equivalent of Task (MET) value (3 for mild, 5 for moderate, 9 for strenuous) and then the individual scores are summed up (Godin 2011; Amireault and Godin 2015). The final score is given in units and depending on the amount of the units, the activity level is shown: active, moderately active or insufficiently active (Godin 2011). Godin and Shephard (1985) confirmed the validity and reliability of the questionnaire.

#### Nicotine dependence

To determine the cigarette dependence of the test persons, the Fagerström Test for Cigarette Dependence was used (FTCD) (Heatherton et al. 1991; Fagerström 2012). The FTCD is a standard instrument for assessing the level of cigarette dependence. The questionnaire consists of six items that evaluate the quantity of cigarette consumption, the compulsion to use, and dependence and are answered by yes or no or multiple-choice. The resulting total score (from 0 to 10) is assigned to a category and indicates the level of cigarette dependence (very low, low, medium, high or very high cigarette dependence) (Svicher et al. 2018). The validity and reliability of the questionnaire has been confirmed (Pomerleau et al. 1994; Etter et al. 1999; Svicher et al. 2018).

#### Cigarette cravings

The strength of desire to smoke (SoD) was evaluated using a single-item question (West et al. 1989). As in the studies of Ussher et al. (2001) and Daniel et al. (2004), participants rated their strength of desire on a seven-point scale (1 = not at all, 4 = somewhat, 7 = extremely).

Cigarette cravings were also measured by the Questionnaire of Smoking Urges (QSU). The 32 items of the questionnaire that are assigned to four different scales, were answered on a scale from 1 (strongly disagree) to 7 (strongly agree). As in the study of Tiffany and Drobes (1991), an analysis of the factors was performed and the items were assigned to Factor 1 or Factor 2 depending on their factor loading. Factor 1 items (15 items) reflected primarily the scales “desire to smoke”, “intention to smoke”, and “anticipation of positive outcome”. Factor 2 (11 items) primarily included items from the scale “relief of withdrawal or negative affect”, with a few items from the previously mentioned three scales also included in Factor 2 (Tiffany and Drobes 1991). The reliability of the English (Tiffany and Drobes 1991) and German version of the questionnaire (Müller et al. 2001) has been confirmed.

#### Assessment of cigarette withdrawal symptoms

To assess cigarette withdrawal symptoms, the Mood and Physical Symptoms Scale (MPSS) (West et al. 1984; West and Russell 1985) was used. The items (1) depression, (2) irritability, (3) restlessness, (4) tension, (5) difficulty concentrating, (6) stress and (7) anxiety were rated on a 5-point scale, ranging from “1” (not at all) to “5” (extremely) (Daniel et al. 2004; West and Hajek 2004). Convergent validity information for the MPSS has been provided by West and Hajek (2004).

#### Assessment of affective responses

The dimension of affective valence was assessed by the feeling scale (FS) (Hardy and Rejeski 1989; Maibach et al. 2020). This single-item rating scale ranges from “ + 5” (very good) to “-5” (very bad), with anchors at “0” (neutral) and at all odd integers. The scale has been used previously in studies measuring the effect of exercise on affect (Frühauf et al. 2016; Stanton et al. 2016). Convergent validity information for the FS has been provided by Hardy and Rejeski (1989) and Van Landuyt et al. (2000).

The dimension of perceived activation was examined by the felt arousal scale (FAS) (Svebak and Murgatroyd 1985; Van Landuyt et al. 2000; Maibach et al. 2020). The single-item rating scale ranges from “1” (low arousal) to “6” (high arousal). The FAS demonstrates convergent validity with other measures of perceived activation (Svebak and Murgatroyd 1985; Van Landuyt et al. 2000).

Ekkekakis and Petruzzello (2002) integrated the affective valence (horizontal dimension) and the perceived activation (vertical dimension) into a circumplex model of affect. This results in four quadrants: high-activation displeasure (e.g. tension, distress) (top left), high-activation pleasure (e.g. energy, vigour) (top right), low-activation displeasure (e.g. tiredness, boredom) (bottom left), and low-activation pleasure (e.g. calmness, relaxation) (bottom right) (Ekkekakis et al. 2011).

### Statistical analyses

Mean (M) and standard deviation (SD) of the data were reported for the presentation of the data. One-way repeated ANOVA was used to examine differences among the three groups in required nicotine abstinence before the three interventions (control measure) and differences in time to smoke the next cigarette after the interventions. Paired *t*-tests were conducted to identify differences in heart rate and perceived exertion (Borg Scale) (control measures) between the IN-EX and OUT-EX group. To examine the differences between the various measurement outcomes of the three groups at different time points, two-way repeated measures analyses of variance (ANOVA) were performed. The ANOVA included two within-subject factors: group (three interventions: control condition, indoor exercise, outdoor exercise) and time (3 to 8 time points depending on assessment instrument). Main effects of group and time, group-by-time interaction and partial ƞ^2^ (ƞ^2^ < 0.06: small effect, ƞ^2^ between 0.06 and 0.14: medium effect, ƞ^2^ > 0.14: large effect) (Cohen 2013) as effect size were calculated. If sphericity verified by Mauchly test was violated, the Greenhouse–Geisser correction was used. Simple contrasts for group variable (CC as reference category for comparison of CC vs. IN-EX and CC vs. OUT-EX, OUT-EX as reference category for comparison of IN-EX vs. OUT-EX) and for time variable (pre-intervention as reference category) were also performed. Data were analyzed using IBM SPSS Statistics (version 26) and statistical significance was declared if *p* < 0.05.

## Results

### Study sample

Eight males and eight females participated in the study. On average, the participants had a low cigarette dependence (FTCD). The participants could be categorized generally as physical active (mean moderate-to-strenuous leisure score index). Two test persons had an insufficient activity level, five were moderately active and nine participants were classified as active (see Table 2 for demographic and smoking related characteristics).

**Table 2:** Demographic, smoking-related characteristics and physical activity of participants

Characteristics (*n* = 16)	Mean (SD)
Age (years)	30.06 (12.3)
BMI (kg/m^2^)	22.08 (2.31)
Years smoking	11.94 (9.96)
Cigarettes per day	14.44 (4.49)
FTCD	4.00 (1.63)
GSLTPAQ (moderate-to-strenuous LSI) Strenuous exercise (min) Moderate exercise (min) Mild exercise (min)	29.88 (18.73) 39.06 (50.93) 34.06 (22.15) 26.88 (14.13)

*BMI*, body mass index; *FTCD,* Fagerström test for cigarette dependence; *GSLTPAQ*, Godin-Shephard Leisure-Time Physical Activity Questionnaire; *LSI*, Leisure Score Index

### Control measures

One-way repeated ANOVA showed no statistically significant difference for the required nicotine abstinence before the three interventions (F (2,30) = 0.072, *p* = 0.931, partial η^2^ = 0.005). Prior to all interventions, participants refrained from smoking for an average of over 11 h (CC: 11.44 h ± 1.59; IN-EX: 11.31 h ± 1.53; OUT-EX: 11.44 h ± 1.55). To ensure that the intensity of IN-EX and OUT-EX is similar, heart rate was measured and perceived exertion was rated using the Borg Scale. No significant difference in heart rate between the IN-EX and the OUT-EX could be seen (t (15) = -0.995, *p* = 0.336). The average heart rate in OUT-EX (119.56 ± 11.42) was slightly higher than in IN-EX (116.69 ± 12.98). The average perceived exertion rated by BORG Scale was significantly higher in IN-EX (11.46 + 1.22) than in the OUT-EX group (10.61 ± 1.05) (t (15) = 2.193, *p* = 0.044).

### Cigarette cravings

**Fig. 1:**
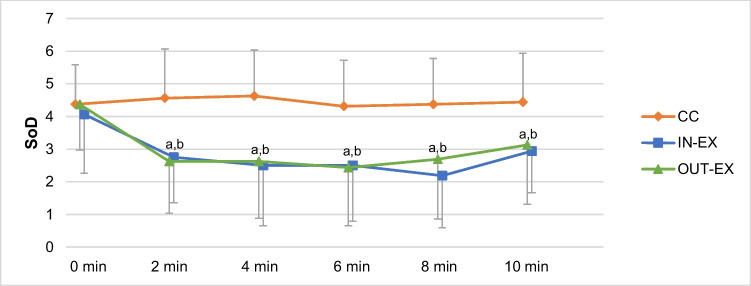
Means of strength of desire to smoke (SoD) for the three groups at different measurement time points (pre-intervention: 0 min, during intervention: 2–8 min, end of intervention: 10 min.). ^a, b^ indicate significant differences of the contrast analyses between two groups at different time points compared to pre-intervention (^a^ CC vs. In-EX, ^b^ CC vs. OUT-EX)

Before the intervention (0 min), all groups showed similar values for the strength of desire to smoke (SoD). In contrast to the CC group, the SoD of the IN-EX and OUT-EX group reduced during (2–8 min) and at the end of intervention (10 min) (see Fig. 1). Significant main effects of group and time and significant group-by-time interaction were found for SoD (see Table 3). Contrast analysis showed significant differences between CC and IN-EX and CC and OUT-EX at all measurement time points during and after the intervention compared to pre-intervention (see Fig. 1). No significant differences could be seen between IN-EX and OUT-EX.

**Table 3:** *P*-values and partial ƞ^2^ for group-by-time interaction and for main effects of group and time of repeated measures ANOVA of different measurements for the three groups over several time points

	*p*-value (partial ƞ^2^)		
Variables	Group	Time	Interaction
SoD	0.002 (0.41)*	< 0.001 (0.44)**	< 0.001 (0.29)**
QSU_F1	0.001 (0.37)*	0.005 (0.29)*	0.007 (0.26)*
QSU_F2	0.007 (0.28)*	0.039 (0.19)*	0.007 (0.25)*
MPSS	0.089 (0.15)	0.001 (0.43)*	0.448 (0.06)
FS	0.870 (0.01)	0.035 (0.20)*	0.022 (0.17)
FAS	0.289 (0.08)	0.214 (0.09)	0.041 (0.14)*
Time to first cigarette	0.323 (0.07)		

*SoD*, strength of desire to smoke; *QSU_F1*, Questionnaire of Smoking Urges Factor 1; *QSU_F2*, Questionnaire of Smoking Urges Factor 2; *MPSS*, Mood and Physical Symptoms Scale; *FS*, Feeling Scale; *FAS*, Felt Arousal Scale. * *p* < 0.05, ** *p* < 0.001

**Fig. 2:**
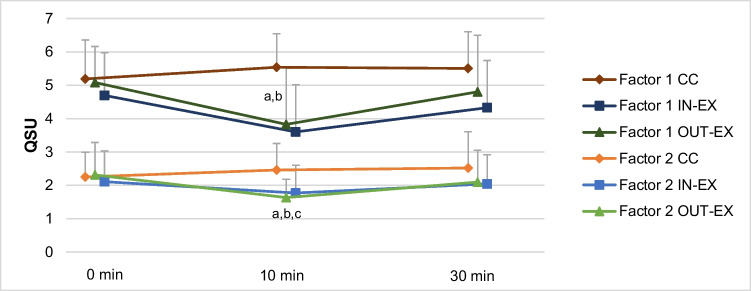
Means of Factor 1 (desire to smoke) (top) and Factor 2 (relief of withdrawal) (bottom) of QSU for the three groups at different measurement time points (pre-intervention: 0 min, end of intervention: 10 min, 2^nd^ follow-up: 30 min.). ^a, b, c^ indicate significant differences of the contrast analyses between two groups at different time points compared to pre-intervention (^a^ CC vs. In-EX, ^b^ CC vs. OUT-EX, ^c^ IN-EX vs. OUT-EX)

Desire to smoke (Factor 1) and relief of withdrawal (Factor 2), assessed by QSU, of both exercise groups decreased after exercise intervention (10 min) and increased slightly after second follow-up (30 min). Scores for both Factors of CC group did not change over time and were higher than in the exercise groups at the end of intervention and at 2^nd^ follow-up (see Fig. 2). Main effects for group and time for Factor 1 and 2 and the group-by-time interaction for both Factors were significant (see Table 3). Contrast analyses of Factor 1 showed significant differences between CC and IN-EX and CC and OUT-EX at the end of intervention compared to pre-intervention. For Factor 2, significant differences between CC and IN-EX, CC and OUT-EX and IN-EX and OUT-EX at the end of intervention compared to pre-intervention could be found.

### Cigarette withdrawal symptoms

**Fig. 3:**
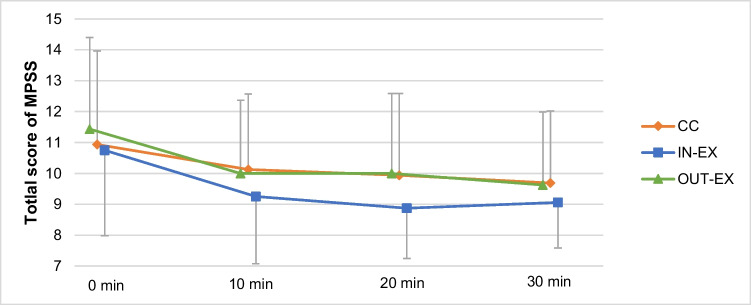
Means of total score of MPSS (Mood and Physical Symptoms Scale) for the three groups at different measurement time points (pre-intervention: 0 min, end of intervention: 10 min, 1^st^ follow-up: 20 min, 2^nd^ follow-up: 30 min)

The total score of all withdrawal symptoms, measured by MPSS, decreased in all three groups after the interventions (see Fig. 3). There was no main effect between groups and no group-by-time interaction for the total score of MPSS (see Table 3). A significant main effect over time could be seen, with the total score of pre-intervention significantly different from the total scores at the other three time points (end of intervention, 1^st^ and 2^nd^ follow-up). No main effect between groups and no group-by-time interaction were found for each of the seven items of the MPSS. Main effects over time were significant for the items “irritability (*p* = 0.047), “tension” (*p* = 0.001), “difficulty in concentration” (*p* = 0.004) and “stress” (*p* < 0.001). A significant reduction of the previous mentioned withdrawal symptoms over time compared to pre-intervention could be seen.

### Affective responses

**Fig. 4:**
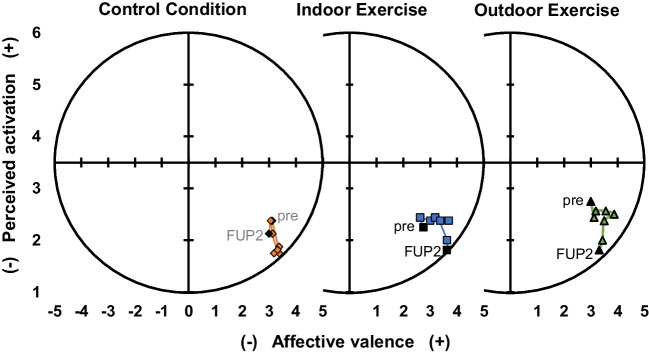
Change in affective valence (*x*-axis) and perceived activation (*y*-axis) within the three groups over different measurement time points (pre, during (2, 4, 6, 8 min), end of intervention, 1^st^ and 2^nd^ follow-up) (the markers for time points “pre” and “2^nd^ follow-up” (FUP2) are filled in black)

Mean values for affective valence, assessed by the Feeling Scale (FS), of all three groups were in a positive range at all measurement time points (see Fig. 4). During exercise interventions (4–8 min), the affective valence increased in the IN-EX and OUT-EX group. In the CC, similar values for affective valence over time could be observed. No main effect for group, but a main effect over time and a significant group-by-time interaction were found (Table 3). Affective valence of IN-EX and OUT-EX was significantly higher than in CC at the end of intervention (10 min) and at both follow-up time points (20 min and 30 min) compared to pre-intervention.

Perceived activation, examined by Felt Arousal Scale (FAS), was rated as low by all groups over time (Fig. 4). In the CC group, perceived activation decreased during intervention (sitting time) and increased after the intervention ended. Both exercise groups showed a higher perceived activation during intervention than the CC group. After the intervention, the perceived activation decreased in both exercise groups at 1^st^ and 2^nd^ follow-up (Fig. 4). No main effects between groups and over time were observed. The group-by-time interaction was significant but contrast analyses showed no significant differences.

Integrating the values of affective valence (horizontal dimension) and perceived activation (vertical dimension) into the circumplex model of affect, we found that the values for all three groups (OUT-EX, IN-EX, CC) over all time points are in the bottom right quadrant (Fig. 4). High affective valence and low perceived activation in the bottom right quadrant indicate low activated pleasure, e.g. calmness and relaxation.

### Time to the first cigarette of the day

A greater delay in smoking the next cigarette after outdoor exercise intervention compared to indoor exercise and control condition could be seen. Participants of the CC and IN-EX group consumed their first cigarette after 16.81 ± 25.46 and 17.00 ± 24.56 min, while participants of the OUT-EX group smoked their first cigarette after 26.19 ± 31.46 min after the intervention. Main effect analysis and contrast analyses of repeated measures ANOVA showed no statistically significant difference in time to next cigarette between the three intervention groups (Table 3).

## Discussion

### Main findings

The aim of our study was to investigate the acute effects of a short bout of outdoor and indoor walking compared to a sitting control condition on cigarette cravings, withdrawal symptoms, affective response and time to the first cigarette in temporarily abstinent smokers. In contrast to the control condition, outdoor and indoor walking for 10 min significantly reduced cigarette cravings during (SoD) and at the end of intervention (SoD and QSU) compared to pre-intervention, but not after 20 min follow-up (QSU). Cigarette withdrawal symptoms (MPSS) significantly decreased over time (end of intervention, 1^st^ and 2^nd^ follow-up) in all three conditions. Exercise was further shown to enhance affective valence and perceived activation compared to the control condition. A significant difference between outdoor and indoor exercise could be found in the Relief of Withdrawal as measured by Factor 2 of QSU, with outdoor walking resulting in lower cigarette cravings than indoor walking at the end of intervention (*p* = 0.02, partial ƞ^2^ = 0.31) compared to pre-intervention.

### Context of the literature

Since this seems to be the first study examining the effects of a single outdoor walking unit compared to indoor walking and sitting control condition in temporary abstinent smokers, a comparison of the results with the existing literature is limited. Nevertheless, similar studies also using a within-subject design and a short indoor walking intervention support the findings of our study that a short bout of exercise reduced cigarette cravings. Scerbo et al. (2010) also found reduced cravings (SoD) during and after 15 min walking on a treadmill compared to a passive control condition. In the study of Janse van Rensburg and Taylor (2008), lower cigarette cravings, measured by two dimensions of the QSU, after 15 min self-paced walking on a treadmill and after 5 and 10 min post-treatment but not after 15 min follow-up was observed, similar to the results in the present study. As in our study, Taylor and Katomeri (2007) reported reduced withdrawal symptoms, evaluated by MPSS, during and after a 15 min indoor walking session. In contrast to our study, which showed no group-by-time interaction and no group differences, the study of Taylor and Katomeri (2007) found significantly lower withdrawal symptoms in the exercise group compared to the control group during and after the treatment. Taylor et al. (2006) observed a significantly higher affective valence (FS) and a higher perceived activation (FAS) at the end of a 15–20 min self-paced 1-mile walk and a higher affective valence (FS) after 10 and 20 min post-treatment compared to a sedentary control condition. In our study, a similar significant effect for affective valence (FS) and similar variations in perceived activation (FAS), for both exercise groups compared to the control group could be seen. Scores for perceived activation were generally lower in our study than in the study of Taylor et al. (2006) over all time points. In contrast to our study, Faulkner et al. (2010) reported a greater delay to smoke the first cigarette after a 10 min indoor walk than after a sitting condition. In the present study, a greater delay to smoke the next cigarette was observed only after the outdoor exercise, whereas the time to smoke the next cigarette was similar after indoor exercise and control condition.

### Interpretation

Despite a broad agreement of the results in the literature with those of the present study, it must be considered that the interventions and samples in all studies differ from each other. Several factors such as the duration and intensity of exercise sessions, duration of smoking abstinence before interventions, socio-demographic factors (e.g. age, gender), smoking history (cigarette dependence, number of cigarettes per day), general fitness level of the sample (low, moderate or high) and general well-being and motivation of the sample, may influence the strength of the relationship between exercise and craving or withdrawal symptoms (Haasova et al. 2016, 2014; Bloom et al. 2012; Tritter et al. 2015). Some studies showed that short bouts of exercise with higher intensity have greater effects on craving and withdrawal symptoms than lower-intensity exercise units (Daniel et al. 2004; Kurti and Dallery 2014; Janse van Rensburg et al. 2013). Daniel et al. (2004) observed this effect in a sample of smokers leading a sedentary lifestyle. In our study, participants were categorized generally as physically active. They showed a low perceived activation (assessed by FAS) during exercise and rated their perceived exertion using BORG Scale as light during indoor and outdoor exercise. The varying intensities between studies could be an explanation for the differing results (Ekkekakis et al. 2011). Scerbo et al. (2010) also reported that after a short running session, the cigarette craving decreased for a longer period compared to a walking session. This would be a possible explanation why the shorter (10 min), moderate walking session in the present study had positive effects on craving after exercise treatment, but not after 20 min post-treatment. Since this effect cannot be confirmed in all studies (Everson et al. 2008), further investigations are needed.

In our study, outdoor and indoor walking sessions showed similar outcomes, while one significant difference in cigarette craving at end of treatment, assessed by Factor 2 of QSU, was observed between outdoor and indoor exercise. A previously suspected additional positive influence of exercise units outside compared to exercise units inside could only be found to a limited extent in our study. An explanation for an additional beneficial effect of natural environments would be the Attention Restoration Theory (ART) by Kaplan (1995). The theory says that relaxing environments, such as nature, provide the opportunity to restore direct attention and recover by allowing nature to provide distance from everyday stress and involuntarily draw attention to calming stimuli in nature (Kaplan 1995; Ohly et al. 2016). In addition, studies reported a positive effect of green exercise (physical activity with a simultaneous exposure to nature (Rogerson et al. 2016)) on affective valence, enjoyment and satisfaction (Lahart et al. 2019), self-esteem, stress and mood (Rogerson et al. 2016) as well as energy and vitality (Fuegen and Breitenbecher 2022; Plante et al. 2006; Ryan et al. 2010). According to ART and the positive impact of green exercise, the results of the present study suggested that the focus of the temporarily abstinent smokers during outdoor walking would be on the natural environment rather than cigarette craving and withdrawal symptoms, which ultimately lead to better well-being, affective response and a longer time to smoke the next cigarette. However, studies investigating the effects of natural environments on cigarette craving and withdrawal symptoms in temporarily abstinent smokers are still lacking. Only Martin et al. (2019) previously conducted a cross-sectional online survey and observed an association between exposure to natural environments and a reduction in craving severity and frequency among individuals who selected an appetitive target for which they regularly craved (e.g. food, chocolate, nicotine, alcohol).

Minor differences between short bouts of outdoor and indoor exercise in the present study could be explained by the fact that partly poor weather conditions (snow, rain, fog, low temperatures) existed during outdoor measurements. Further, outdoor walking took place on flat gravel paths in urban green spaces. The literature has shown that environmental factors such as weather, daylight and location can have various influences on well-being (MacKerron and Mourato 2013). MacKerron and Mourato (2013) observed greater happiness in study participants who spend time outdoors in all green and natural environments than in urban environments. In the study of Pasanen et al. (2018), some aspects of restoration were greater in natural environments than in indoor and built outdoor (streets, sports fields) settings.

Nevertheless, both exercise conditions, outdoor and indoor walking, showed greater effects on cigarette cravings and affective response than the control condition. The underlying mechanisms that may explain why exercise alleviates cigarette cravings have not been fully explored and are unclear (Ussher et al. 2019). However, possible mechanisms, such as biological, affect and cognitive hypotheses, may contribute to clarification (Roberts et al. 2012). There are some biological changes during smoking and exercising regarding the stimulation of the central nervous system (Ussher et al. 2019; Roberts et al. 2012). Functional Magnetic Resonance Imaging (fMRI) scans in the study of Janse van Rensburg et al. (2009) showed, that areas of the brain, which are normally activated by smoking cues, were less active after a moderate exercise session (Ussher et al. 2019; Ledochowski et al. 2013). The Hypo Frontality Theory (THFT) supports this finding. THFT assumes that physical activity primarily activates sensory, motor and autonomic brain regions and reduces activation in other areas related to reward processing and visuospatial attention (Ledochowski et al. 2013; Roberts et al. 2012). As a result, the perception of negative emotional states, cigarette cravings and withdrawal symptoms (stress, anxiety, tension, restlessness) is reduced during exercising (Ledochowski et al. 2013). In addition, both smoking and exercise lead to the release of various neurotransmitters such as adrenaline, cortisol and ß-endorphin (West 2017; Yildiz 2004; Roberts et al. 2012). Despite a change in cortisol levels during exercise, studies have not yet found an association with changes in cigarette cravings (Ussher et al. 2019; Roberts et al. 2015; Scerbo et al. 2010; Jesus and Prapavessis 2018). However, some studies observed a correlation between changes in noradrenaline levels during exercise and reduced cigarette cravings (Pomerleau et al. 1987; Roberts et al. 2015). Moreover, it has been suggested that exercise may influence cognitive demands and that the attention during exercise is focused on movements and bodily sensations, which may distract from the desire to smoke (Roberts et al. 2015; Ussher et al. 2019). However, studies have not yet been able to prove this hypothesis (Ledochowski et al. 2013; Daniel et al. 2006; Ussher et al. 2019). There is also evidence, that well-being and affective response are positively influenced by exercise and a higher positive affect is associated with a decreased desire to smoke and reduced withdrawal symptoms (Roberts et al. 2015; Everson et al. 2008; Elibero et al. 2011).

### Limitations

This study had several strengths but was also limited by some aspects. Although the average time participants refrained from smoking before interventions was according to literature (> 10 h) (Roberts et al. 2012), no objective measurement to measure expired carbon monoxide levels was used to verify participants’ self-reported abstinence time (e.g. Smokerlyzer). The control variables heart rate (objective assessment) and perceived exertion (subjective assessment using BORG scale) were used to verify that the intensity of the outdoor and indoor exercise sessions was comparable. However, the two control variables differed slightly: While the OUT-EX group had a higher average heart rate than the IN-EX group (no significant difference), the indoor group rated the perceived exertion rate during walking as more strenuous than the outdoor group (significant difference). As in other studies (Taylor et al. 2005; Faulkner et al. 2010), participants in the present study, who had different fitness levels prior to study inclusion, were able to self-determine their walking speed, but were instructed to walk as if they were late for an appointment. It may be that this instruction is easier to implement and control during the outdoor exercise session because it is closer to reality, resulting in a higher heart rate during OUT-EX than IN-EX. One possible reason why the perceived exertion level was slightly lower but the heart rate higher in the OUT-EX group than in the IN-EX group could be that the natural environment had a potential impact on self-perception. For example, during the outdoor walking session, participants may have perceived the exercise session as less strenuous due to distractions in nature and positive emotions from the natural environment (Fuegen and Breitenbecher 2022; Kaplan 1995). For a more accurate determination of participants’ exercise intensity in our study and the classification of exercise intensity into low, moderate or high, a maximal endurance test could have been conducted in advance (very time-consuming), or the exercise intensity could have been determined using a formula like in the studies of Conklin et al. (2017) and Elibero et al. (2011).

In addition, although the order of interventions was randomized for participants, participants might have been aware of the expected benefits of exercise, which could have biased the outcomes (Roberts et al. 2012). It should also be considered, that the use of self-reported questionnaires could lead to a reporting bias (McGauran et al. 2010). Furthermore, the sample size in our study was very small, which might mean that statistical analyses will not show significant results even if effects were present. Moreover, weather conditions were not controlled for and thus resulted in some participants experiencing poor weather conditions during the outdoor intervention which could have influenced the results. For this reason, further studies are needed that also examine the effect of different environmental conditions during outdoor exercise sessions on craving and withdrawal symptoms.

## Conclusion

To the best of our knowledge, this was the first study investigating the acute effects of a short bout of outdoor and indoor walking compared to a sitting control condition on cigarette cravings, withdrawal symptoms, affective response and time to the first cigarette in temporarily abstinent smokers. The study added to existing evidence that walking outdoors and indoors for 10 min could be helpful in reducing cigarette cravings, withdrawal symptoms and increasing affective response for a limited period of time. Brief outdoor exercise resulted in significantly lower cigarette cravings than indoor exercise at the end of intervention. A greater but non-significant delay in smoking the next cigarette after outdoor exercise was also observed compared to indoor exercise. Since short walking bouts outdoors at a self-paced walking speed are easy to implement in everyday life and have further additional health benefits, this intervention should be considered as an important adjunct in smoking cessation programs and promoted among smokers who want to quit smoking as well as a behavioral instrument to overcome cigarette cravings and withdrawal symptoms. Further studies using larger sample sizes are needed to verify the additional beneficial outdoor effects and also investigating whether different environmental conditions (weather, temperature, location) lead to different effects on craving, withdrawal symptoms and well-being. In addition, it is unclear whether the reduction in cigarette craving and withdrawal symptoms among abstinent smokers was due to the physical activity itself, the natural environment in which the outdoor walking took place or both. Therefore, further studies with larger sample sizes and, for example, two intervention groups (indoor walking and outdoor walking) and two control groups (indoor sitting and outdoor sitting) are needed to determine which components are most likely to reduce cigarette craving and withdrawal symptoms.
